# Insulin Is a Key Modulator of Fetoplacental Endothelium Metabolic Disturbances in Gestational Diabetes Mellitus

**DOI:** 10.3389/fphys.2016.00119

**Published:** 2016-03-31

**Authors:** Luis Sobrevia, Rocío Salsoso, Bárbara Fuenzalida, Eric Barros, Lilian Toledo, Luis Silva, Carolina Pizarro, Mario Subiabre, Roberto Villalobos, Joaquín Araos, Fernando Toledo, Marcelo González, Jaime Gutiérrez, Marcelo Farías, Delia I. Chiarello, Fabián Pardo, Andrea Leiva

**Affiliations:** ^1^Cellular and Molecular Physiology Laboratory, Division of Obstetrics and Gynecology, Faculty of Medicine, School of Medicine, Pontificia Universidad Católica de ChileSantiago, Chile; ^2^Faculty of Medicine and Biomedical Sciences, University of Queensland Centre for Clinical Research, University of QueenslandHerston, QLD, Australia; ^3^Department of Physiology, Faculty of Pharmacy, Universidad de SevillaSeville, Spain; ^4^Department of Basic Sciences, Faculty of Sciences, Universidad del Bío-BíoChillán, Chile; ^5^Vascular Physiology Laboratory, Department of Physiology, Faculty of Biological Sciences, Universidad de ConcepciónConcepción, Chile; ^6^Group of Research and Innovation in Vascular Health (GRIVAS-Health)Chillán, Chile; ^7^Cellular Signaling and Differentiation Laboratory, Health Sciences Faculty, Universidad San SebastiánSantiago, Chile

**Keywords:** insulin, gestational diabetes, endoplasmic reticulum stress, angiogenesis, lipids, placenta, endothelium

## Abstract

Gestational diabetes mellitus (GDM) is a disease of the mother that associates with altered fetoplacental vascular function. GDM-associated maternal hyperglycaemia result in fetal hyperglycaemia, a condition that leads to fetal hyperinsulinemia and altered L-arginine transport and synthesis of nitric oxide, i.e., endothelial dysfunction. These alterations in the fetoplacental endothelial function are present in women with GDM that were under diet or insulin therapy. Since these women and their newborn show normal glycaemia at term, other factors or conditions could be altered and/or not resolved by restoring normal level of circulating D-glucose. GDM associates with metabolic disturbances, such as abnormal handling of the locally released vasodilator adenosine, and biosynthesis and metabolism of cholesterol lipoproteins, or metabolic diseases resulting in endoplasmic reticulum stress and altered angiogenesis. Insulin acts as a potent modulator of all these phenomena under normal conditions as reported in primary cultures of cells obtained from the human placenta; however, GDM and the role of insulin regarding these alterations in this disease are poorly understood. This review focuses on the potential link between insulin and endoplasmic reticulum stress, hypercholesterolemia, and angiogenesis in GDM in the human fetoplacental vasculature. Based in reports in primary culture placental endothelium we propose that insulin is a factor restoring endothelial function in GDM by reversing ERS, hypercholesterolaemia and angiogenesis to a physiological state involving insulin activation of insulin receptor isoforms and adenosine receptors and metabolism in the human placenta from GDM pregnancies.

## Introduction

A large number of pregnant women are diagnosed with gestational diabetes mellitus (GDM), a disease that appears in pregnancy, courses with maternal hyperglycaemia and leads to fetal hyperglycaemia and hyperinsulinemia [American Diabetes Association (ADA), 2015]. These women are subjected to a calories-controlled diet with the final goal of reducing glycaemia to values as those in normal pregnancies. Interestingly, even when the mother and newborn from GDM under diet protocol show normal glycaemia at birth, metabolic alterations reducing the reactivity of the placenta and umbilical cord vessels (i.e., fetoplacental vasculature) are still seen (Sobrevia et al., 2015). GDM results in reduced fetoplacental vascular dilation in response to insulin or adenosine (an endogenous vasodilator nucleoside) via mechanisms including altered expression of adenosine receptors (ARs) and/or insulin receptors forms A (IR-A) and B (IR-B), L-arginine and adenosine membrane transport and transporters expression in the human fetoplacental macrovascular and microvascular endothelium. GDM results in higher synthesis of nitric oxide (NO) and expression of the endothelial NO synthase (eNOS), and L-arginine transport (the substrate for eNOS), a phenomenon attributed to ARs activation by adenosine in primary cultures of human umbilical vein endothelial cells (HUVECs) and human placental microvascular endothelial cells (hPMECs) (Westermeier et al., 2011, 2015a; Salomón et al., 2012). Insulin reverses this disease's associated alterations, requiring ARs activation (Guzmán-Gutiérrez et al., 2016) via selective activation of IR-A and/or IR-B, and ARs activation leading to IR-A-associated mitogenic or IR-B-associated metabolic phenotype in these cell types (Westermeier et al., 2015a,b).

GDM triggers a variety of stressor signals leading to abnormal function of intracellular structures, including the endoplasmic reticulum (ER). GDM increases the activity of molecules associated with ER stress (ERS) (Marciniak and Ron, 2006). Insulin signal is altered in ERS (Ron and Walter, 2007; Sáez et al., 2014) and GDM (Sáez et al., 2014), a condition ending in insulin resistance. Interestingly, ERS and GDM course with altered activity of several transcription factors, such as the pro-apoptotic transcription factor growth arrest and DNA damage 153 (GADD153) (also referred as C/EBP homologous protein 10 or CHOP). Even when is known that human CHOP (hCHOP) is activated by NO leading to reduced expression of *SLC291A* gene [for human equilibrative nucleoside transporter 1 (hENT1)] (Farías et al., 2010), and that hCHOP activity is modulated by insulin (Sáez et al., 2014), nothing is clear regarding a potential involvement of ARs and/or IRs in this phenomenon. On the other hand, pregnant women coursing with supraphysiological hypercholesterolemia show altered fetoplacental NO-dependent and L-arginine transport-dependent vascular reactivity when plasma level of total cholesterol (TCh) is >280 mg/dL (Leiva et al., 2015). However, the vascular effect of maternal dyslipidaemia, or whether ERS and changes in cell signaling and/or expression of ARs or IRs in these alterations is not yet reported. Since ERS and maternal dyslipidaemia modulate angiogenesis (Gutiérrez et al., 2016), and because GDM associates with placental endothelium and trophoblast release of pro-angiogenic factors, dysfunction of these cell types in ERS or maternal dyslipidaemia could result in accelerated angiogenesis.

Thus, in this review, we have emphasized the possibility that an abnormal metabolic state in pregnancy, as seen in GDM, leads to fetoplacental disturbances resulting in ERS, uncontrolled angiogenesis, or lipid metabolism. The involvement of insulin modulation of human fetoplacental vasculature function and its consequences in these phenomena are discussed.

## Gestational diabetes mellitus

GDM is a disease that first appears or is identified during pregnancy [American Diabetes Association (ADA), 2015], associates with abnormal vascular function of the placenta (Colomiere et al., 2009; Haas, 2014), and leads to deleterious consequences to the fetus development and growth as well as to the health of the mother (König et al., 2014; Lappas, 2014). The incidence of this disease of pregnancy is ~7% worldwide [Ferrara et al., 2004; Dabelea et al., 2005; American Diabetes Association (ADA), 2015]. With the goal of reaching maternal glycaemia in a normal range, so to avoid deleterious consequences of hyperglycaemia in the growing fetus, patients diagnosed with GDM are subjected to controlled diet (plus a suggested routine of exercise) or treated with insulin [i.e., insulin therapy; Verier-Mine, 2010; American Diabetes Association (ADA), 2015; Sobrevia et al., 2015].

GDM causes an abnormal supply of nutrients (e.g., D-glucose, amino acids) to the fetus [Leach, 2011; American Diabetes Association (ADA), 2015; Sobrevia et al., 2015], a phenomenon that depends on the fetoplacental vascular tone and blood flow. Since the distal segment of the umbilical cord and the placenta lack of innervation (Fox and Khong, 1990; Marzioni et al., 2004), local regulation of the vascular tone results from the synthesis, release, and bioactivity of endothelium-derived vasodilators and vasoconstrictors (Pearson and Gordon, 1985; Olsson and Pearson, 1990). The endothelium of the human fetoplacental vasculature is a monolayer directly facing fetal blood and corresponds to the epithelium underlying the syncytiotrophoblast layer (Burton and Jauniaux, 2015). Thus, the endothelium is the first target for a variety of circulating molecules in the fetal blood. Equally, it is exposed to maternal blood molecules and/or their metabolites that cross or are released from the syncytiotrophoblast. Interestingly, the level of the endogenous nucleoside adenosine, a potent vasodilator, is increased in human umbilical whole blood (Maguire et al., 1998; Westermeier et al., 2011), or umbilical vein blood (Westermeier et al., 2015a), but not in umbilical arteries blood (Salomón et al., 2012), in GDM pregnancies where the mother was under diet compared with normal pregnancies. These findings were paralleled by reduced uptake of adenosine in HUVECs and hPMECs. Thus, an altered adenosine handling (e.g., uptake, release, metabolism) by the microvascular and macrovascular endothelium of the fetoplacental unit in GDM was proposed (Sobrevia et al., 2015; Westermeier et al., 2015b).

GDM also associates with increased expression of *SLC7A1* gene coding for hCAT isoform 1 (hCAT-1) and higher L-arginine/NO signaling pathway activity in HUVECs (Guzmán-Gutiérrez et al., 2016). These alterations result from activation of the L-arginine transport and NO synthesis as a result of activation of ARs (i.e., ALANO signaling pathway) in this cell type from GDM. The extracellular level of adenosine is mainly, if not only, maintained by the activity of the Na^+^-independent hENT1 and hENT2 in the fetoplacental endothelium (Sobrevia et al., 2015). Altered expression and/or activity of these membrane transporters result in changes in extracellular adenosine concentration, thus altering its normal and broad modulatory actions on cell function (Fredholm et al., 2011; Fredholm, 2014; Verkhratsky and Burnstock, 2014; Burnstock, 2016).

### Insulin and adenosine receptors in GDM

The insulin receptor splice variants IR-A and IR-B result from the absence or presence of a 12-amino acid segment (encoded by exon 11) at the C-terminal of the extracellular α-subunit (Westermeier et al., 2015b). IR-A and IR-B are differentially expressed, including the fetoplacental tissue, and signal via preferential mechanisms depending on their binding affinities for insulin, receptors internalization, receptors recycling time, and intracellular signaling (Westermeier et al., 2015b). It is reported that insulin via activation of IR-A and/or IR-B modulates the expression and activity of hCAT-1, eNOS, hENT1, and hENT2 in HUVECs and hPMECs (Table 1). These findings show that insulin modulates L-arginine and adenosine transport reversing the GDM-associated alterations in these mechanisms to values in human fetoplacental endothelium from normal pregnancies.

**Table 1:** **Effect of insulin on human fetoplacental vasculature in GDM**.

**Cell/tissue**	**Insulin receptor isoform**	**Effect of GDM**	**Insulin effect**	**References**
HUVECs	–	Lower overall adenosine transport	Increase	Westermeier et al., 2011
HUVECs	IR-A	Lower hENT1 activity	Increase	Westermeier et al., 2011, 2015a
HUVECs	–	Higher NOS activity	Decrease	Westermeier et al., 2011
HUVECs	–	Higher eNOS protein abundance	Decrease	Westermeier et al., 2011
HUVECs	–	Higher eNOS phosphorylation in Ser^1177^	Decrease	Westermeier et al., 2011
HUVECs	IR-A	Lower hENT1 protein abundance	Increase	Westermeier et al., 2011, 2015a
HUVECs	IR-A	Lower hENT1 mRNA expression	Increase	Westermeier et al., 2011, 2015a
HUVECs	IR-A, IR-B	Lower *SLC29A1* promoter activity	Increase	Westermeier et al., 2011, 2015a
HUVECs	–	Higher adenosine concentration	Decrease	Westermeier et al., 2011
HUVECs	–	Higher IR-A mRNA	Decrease	Westermeier et al., 2011
HUVECs	IR-A	Lower hENT1 activity	Increase	Westermeier et al., 2015a
HUVECs	IR-A	Lower insulin receptor β subunit phosphorylation	Increase	Westermeier et al., 2015a
HUVECs	–	Lower plasma membrane hENT1 protein abundance	Increase	Westermeier et al., 2015a
HUVECs	IR-A	Higher p44/42^mapk^ phosphorylation	Decrease	Westermeier et al., 2015a
HUVECs	IR-B	Unaltered Akt phosphorylation	Increase	Westermeier et al., 2015a
HUVECs	–	Lower L-leucine incorporation	Increase	Sobrevia et al., 1998
HUVECs^*^	–	Lower L-leucine incorporation	Unaltered	Sobrevia et al., 1998
HUVECs	–	Lower thymidine incorporation	Unaltered	Sobrevia et al., 1998
HUVECs^*^	–	Lower thymidine incorporation	Unaltered	Sobrevia et al., 1998
HUVECs	–	Higher TPP^+^ influx	Decrease	Sobrevia et al., 1998
HUVECs^*^	–	Higher TPP^+^ influx	Unaltered	Sobrevia et al., 1998
HUVECs	–	Higher L-lysine transport	Decrease	Sobrevia et al., 1998
HUVECs^*^	–	Higher L-lysine transport	Unaltered	Sobrevia et al., 1998
HUVECs	–	Higher L-arginine transport	Decrease	Sobrevia et al., 1998
HUVECs^*^	–	Higher L-arginine transport	Unaltered	Sobrevia et al., 1998
HUVECs	–	Higher cGMP accumulation	Decrease	Sobrevia et al., 1998
HUVECs^*^	–	Higher cGMP accumulation	Unaltered	Sobrevia et al., 1998
HUVECs	–	Lower 6-keto-PGF_1α_ synthesis	Unaltered	Sobrevia et al., 1998
UV rings	–	Lower relaxation	Increase	Westermeier et al., 2011
hPMECs	–	Lower overall adenosine transport	Increase	Salomón et al., 2012
hPMECs	–	Lower hENT1 activity	Unaltered	Salomón et al., 2012
hPMECs	IR-A, IR-B	Lower hENT2 activity	Increase	Salomón et al., 2012
hPMECs	–	Lower hENT1 protein abundance	Unaltered	Salomón et al., 2012
hPMECs	IR-A, IR-B	Lower hENT2 protein abundance	Increase	Salomón et al., 2012
hPMECs	–	Lower hENT1 mRNA expression	Unaltered	Salomón et al., 2012
hPMECs	IR-A, IR-B	Lower hENT2 mRNA expression	Increase	Salomón et al., 2012
hPMECs	IR-A, IR-B	Lower *SLC29A2* promoter activity	Increase	Salomón et al., 2012
hPMECs	IR-A	Lower p44/42^mapk^ phosphorylation	Increase	Salomón et al., 2012
hPMECs	IR-B	Lower Akt phosphorylation	Increase	Salomón et al., 2012
hPMECs	IR-A	Lower IR-A mRNA expression	Increase	Salomón et al., 2012
hPMECs	IR-B	Increased IR-B mRNA expression	Decrease	Salomón et al., 2012
fpECs	–	Increased MT1-MMP protein abundance	Increase	Hiden et al., 2012
fpECs	–	Effect not reported on Akt phosphorylation	Increase	Hiden et al., 2012
fpECs	–	Effect not reported on p44/42^mapk^ phosphorylation	Increase	Hiden et al., 2012
HUASMCs	–	Increased overall adenosine transport	Decrease	Aguayo et al., 2001
HUASMCs	–	Increased cGMP accumulation	Unaltered	Aguayo et al., 2001
HUASMCs	–	Increased NOS activity	Unaltered	Aguayo et al., 2001
HUASMCs	–	Lower cAMP accumulation	Increase	Aguayo et al., 2001
Placental tissue	–	Unaltered IRS-1 protein expression	Decrease	Colomiere et al., 2009
Placental tissue	–	Increased IRS-2 protein expression	Unaltered	Colomiere et al., 2009
Placental tissue	–	Unaltered PI3-K p85α protein expression	Decrease	Colomiere et al., 2009
Placental tissue	–	Unaltered GLUT-1 protein expression	Increase	Colomiere et al., 2009
Placental tissue	–	Unaltered IRS-2 mRNA expression	Increase	Colomiere et al., 2009
Placental tissue	–	Unaltered PI3-K p85α mRNA expression	Decrease	Colomiere et al., 2009
Placental tissue	–	Unaltered GLUT-1 mRNA expression	Increase	Colomiere et al., 2009
Placental tissue	–	Unaltered GLUT-4 mRNA expression	Decrease	Colomiere et al., 2009

HUVECs, human umbilical vein endothelial cells; hPMEC, human placental microvascular endothelial cells; HUASMCs, human umbilical artery smooth muscle cells; fpEC, feto-placental endothelial cells; hENT1, human equilibrative nucleoside transporters 1; hENT2, human equilibrative nucleoside transporters 2; SLC29A1, solute carrier family 29 (equilibrative nucleoside transporter) member 1; NOS, nitric oxide synthase; eNOS, endothelial NOS; Ser, Serine; IR-A, insulin receptor A; IR-B, insulin receptor B; IRS-1, insulin receptor substrate 1; IRS-2, insulin receptor substrate 2; cGMP, cyclic guanosine monophosphate; cAMP, cyclic adenosine monophosphate; PI3-K, phosphatidylinositol 3-kinase; p44/42^mapk^, p42/44 mitogen-activated protein kinase; TPP^+^, tetra[^3^H]phenylphosphonium; MT1-MMP, membrane-type matrix metalloproteinase 1; GLUT-1, glucose transporter type 1; GLUT-4, glucose transporter type 4.

* Cells were treated with 25 mmol/L D-glucose for 24 h in vitro.

Adenosine causes relaxation of human umbilical vein rings (*in vitro*) from normal pregnancies requiring activation of endothelial ARs with a major contribution of A_2A_ adenosine receptors (A_2A_AR) compared with A_1_ (A_1_AR), A_2B_ (A_2B_AR), or A_3_ (A_3_AR) isoforms (Westermeier et al., 2011; Guzmán-Gutiérrez et al., 2016). ARs are expressed in HUVECs (Wyatt et al., 2002) and hPMECs (Escudero et al., 2008), of which A_2A_AR predominates. Despite the increase in ALANO signaling pathway in response to A_2A_AR activation by adenosine in HUVECs from normal or GDM pregnancies, characterization of ARs-associated cell signaling and a role for other than A_2A_AR in this phenomenon are still unknown (Fredholm, 2014; Verkhratsky and Burnstock, 2014; Sobrevia et al., 2015; Burnstock, 2016). We recently showed that A_1_AR expression and activation are required for insulin reversal of GDM-increased hCAT-1-mediated L-arginine transport and NO synthesis in HUVECs (Guzmán-Gutiérrez et al., 2016). However, nothing is reported addressing the possibility of a potential differential expression and/or cell signaling of ARs accounting for these effects in the fetoplacental endothelium from normal pregnancies, or in mothers with GDM [Verier-Mine, 2010; American Diabetes Association (ADA), 2015; Sobrevia et al., 2015].

Interestingly, an increase in the extracellular level of adenosine correlates with cardiovascular pathophysiological factors, including shear stress. Since one cellular mechanism explaining the increased level of adenosine and reduced hENT1-mediated transport in HUVECs from GDM is a higher activity of hCHOP transcription factor which is one key component of ERS response in this disease (Farías et al., 2010). Even more, GDM-associated increase in hCHOP activity is attenuated by insulin in HUVECs. Thus, ERS is an abnormal metabolic condition associated with GDM, but the role of insulin in this phenomenon is unclear.

## Endoplasmic reticulum stress

The normal function of ER is essential for the synthesis and processing of secretory and membrane proteins, lipid biosynthesis, and calcium storage (Marciniak and Ron, 2006). The ER is highly sensitive to alterations in cellular environmental changes and acts as a quality control station allowing the transit of correctly folded proteins and retaining unfolded or misfolded proteins (Hetz et al., 2015). Thus, ER plays a key role in the general cellular response to nutrient overload or deprivation, the abnormal increase in the synthesis of secretory proteins, expression of mutant, or misfolded proteins and microbial infections, among others (Ron and Walter, 2007). These “stressor signals” disrupts ER homeostasis and accumulates unfolded proteins in the ER lumen, a phenomenon referred as ERS. In order to adapt ER function to this stress condition, the unfolded protein response (UPR) or ERS are activated (Marciniak and Ron, 2006; Zhang and Kaufman, 2006; Ron and Walter, 2007; Hetz et al., 2015).

An integrated ERS response involves transcriptional activation of multiple genes mediated by inositol-requiring enzyme 1 α (IRE1α) and activating transcription factor 6 (ATF6). It leads to a general decrease in protein translation and selective expression of specific mRNAs mediated by double-stranded RNA-dependent protein kinase (PKR)-like ER-associated kinase (PERK) (Marciniak and Ron, 2006). Thus, IRE1α, ATF6, and PERK are referred as ERS sensors. Interestingly, ERS response is also associated with activation of multiple transcription factors, including X-box binding protein-1 (XBP1) and activating transcription factor 4 (ATF4), regulating the expression of genes involved in the final adaptive effects of UPR. Under normal conditions, the UPR pathway functions as a physiological adaptive mechanism (Hetz et al., 2015). However, when a primary stimulus is too persistent or severe, the ERS response could lead to irreversible cell damage and programed cell death through hCHOP stimulation (Marciniak and Ron, 2006; Zhang and Kaufman, 2006; Ron and Walter, 2007). ERS response is thus critical for a normal cellular homeostasis, and plays relevant roles in the pathogenesis of multiple diseases such as GDM, DMT1, DMT2, obesity, inflammation, cardiovascular disorders, viral infections, neurodegeneration, and cancer (Marciniak and Ron, 2006; Zhang and Kaufman, 2006; Ron and Walter, 2007; Díaz-Villanueva et al., 2015; Salvadó et al., 2015).

### ERS in GDM

Since the UPR is a general homeostatic mechanism for cellular defense, GDM-associated alterations could be different in maternal and fetal tissues. The multiple functional alterations described in human fetal endothelial cells from pregnancies with GDM include reduced expression and activity of hENT1 in HUVECs (Farías et al., 2010), likely due to ARs activation by adenosine (Burnstock, 2002, 2016; Fredholm, 2014). Interestingly, hCHOP, a key component of ERS response, act as an NO-dependent transcriptional repressive factor of *SLC29A1* (for hENT1) expression in HUVECs from GDM pregnancy. An increase in the expression and activity (i.e., DNA binding) of hCHOP has been implicated in the apoptotic branch of UPR, especially when the stressor stimuli overcome the compensatory capacity of the ER in this phenomenon (Eizirik et al., 2008; Hotamisligil, 2010). Thus, GDM-associated alterations in adenosine transport in human fetal endothelial cells could be partially explained by activation of the ER-related hCHOP transcription factor. Indeed, increased expression of hCHOP in HUVECs from GDM pregnancies could be considered as an index of cellular stress. These findings are consistent with the detection of hCHOP induction in cells exposed to an elevated extracellular level of homocysteine (Outinen et al., 1999), suggesting its involvement in endothelial dysfunction caused by hyperhomocysteinemia in patients with diabetes mellitus (Austin et al., 2004; Ndrepepa et al., 2006; Sharma et al., 2006). Thus, expression of the ERS marker hCHOP in HUVECs from GDM pregnancies rise the possibility that UPR is active in this cell type and eventually in other maternal and fetal tissues in this pathology. To date, a key component of the ERS pathway referred as apoptosis signal-regulating kinase 1 (ASK1) is activated in mothers with diabetes mellitus, and plays a causal role in a defective neural tube formation (Wang et al., 2015). In addition, ERS response may also be involved in maternal diabetes-associated cardiac malformations, affecting the embryonic cardiogenesis period (Zhao, 2012).

### Insulin and ERS in GDM

**Figure 1:**
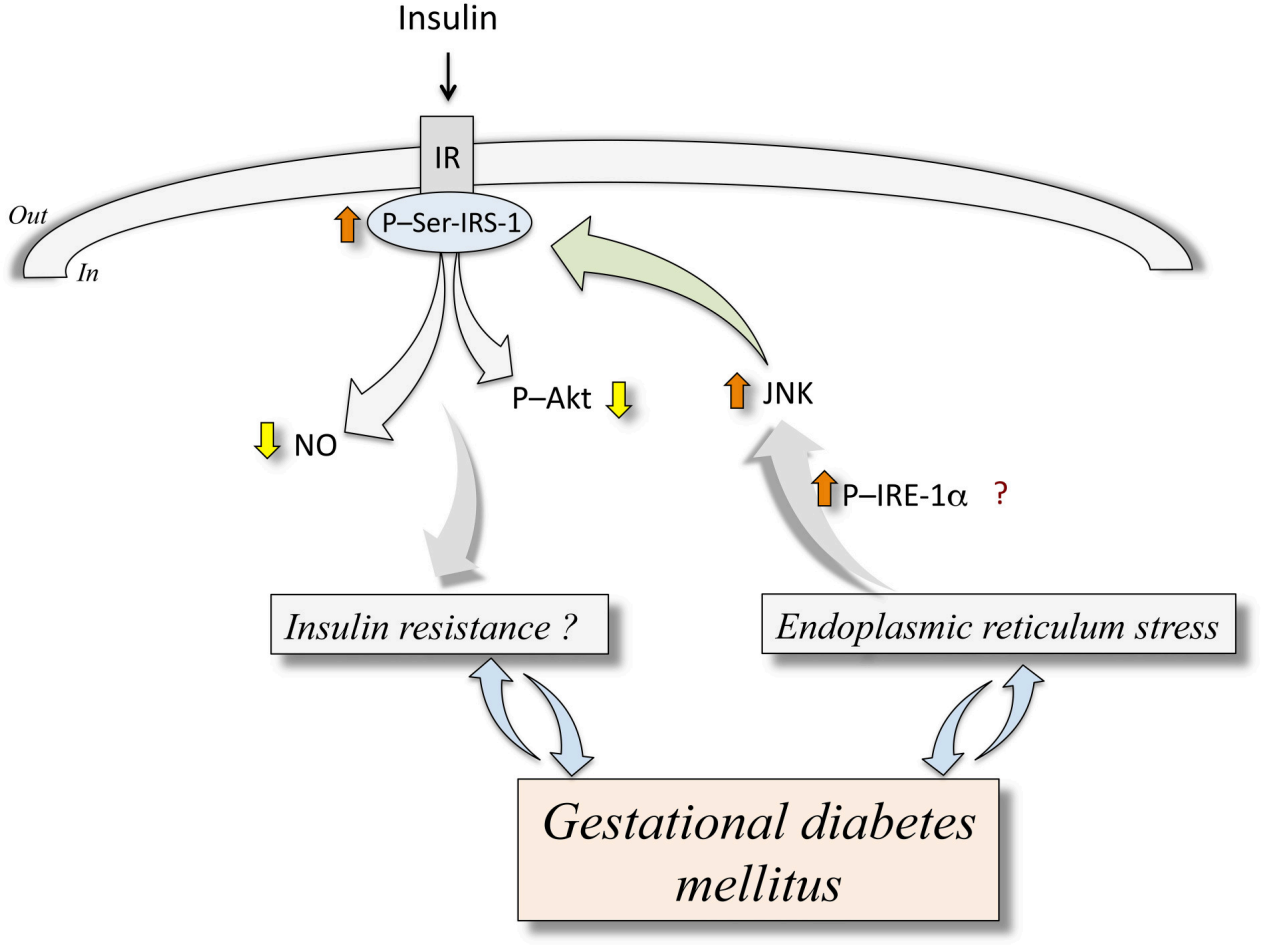
**Endoplasmic reticulum stress and abnormal insulin signaling in human fetoplacental endothelium from gestational diabetes mellitus**. Gestational diabetes mellitus is a disease that associates with endoplasmic reticulum stress (ERS). The latter is an abnormal metabolic condition that could (?) lead to increased (⇧) phosphorylation of inositol-requiring enzyme 1α (P-IRE-1α) resulting in higher c-Jun N-terminal kinase (JNK) activity. This phenomenon causes phosphorylation of insulin receptor substrate 1 at serine^307^ (P-Ser-IRS-1) ending in lower insulin receptor (IR)-associated cell signaling in response to insulin. This altered response to insulin results in reduced (⇩) synthesis of nitric oxide (NO) and phosphorylation of protein kinase B/Akt (P-Akt). These mechanisms are proposed to be potentially involved in insulin resistance (*Insulin resistance?*) in the human fetoplacental endothelium. At present, it is unclear whether GDM causes insulin resistance or ERS, or are these abnormal metabolic conditions that result in GDM clinical manifestations. Composed from information reported by Ozcan et al. (2004, 2006), Taniguchi et al. (2006), Eizirik et al. (2008), Hotamisligil (2010), Sáez et al. (2014), Westermeier et al. (2011, 2015a).

The ERS response associates with a development of insulin resistance in the context of obesity or DMT2 (Ozcan et al., 2004, 2006; Sáez et al., 2014). The c-Jun N-terminal kinase (JNK) is activated through an IRE-1α-dependent phosphorylation in response to ERS in endothelial cells from humans with diabetes mellitus and in animal models of diabetes (Eizirik et al., 2008; Hotamisligil, 2010; Figure 1). Activation of JNK leads to phosphorylation of serine^307^ on insulin receptor substrate 1 (IRS-1), thus inhibiting insulin signaling pathway, a condition that turns into a stage of insulin resistance due to defective downstream signaling, including reduced protein kinase B/Akt (Akt) activation and NO synthesis (Taniguchi et al., 2006; Hotamisligil, 2010). Additionally, ERS correlates with activation of an inflammatory response inducing interleukin 1α (IL-1α) and interleukin 1β (IL-1β) secretion in adipose tissue of pregnant women that are obese or with GDM (Liong and Lappas, 2015). Since IL-1β is a major contributor to the pathophysiology of obesity in pregnancy and GDM (Colomiere et al., 2010; Liong and Lappas, 2015), inhibition of ERS-induced IL-1β synthesis may be a potential therapeutic approach to improve pregnancy complications associated with maternal obesity and GDM, including altered insulin resistance. Thus, a growing body of evidence addresses that ERS may be activated in maternal and fetal tissues in GDM pregnancy. Since insulin signaling could be under modulation by key components of ERS pathway, it is possible that both, maternal and fetal insulin response may be reduced under conditions of ERS. Looking for eventual ERS alleviating interventions in pregnancy may contribute to the prevention of GDM-associated, ERS-related alterations of insulin biological effects.

## Placental angiogenesis

One of the main steps in the placenta formation is the development of its highly structured and specialized net of blood vessels. Formation of placental blood vessels occurs with (*i*) vasculogenesis, which begins at the end of the third week of gestation and corresponds to the formation of the first vascular plexus from pluripotent progenitor cells which then differentiate into endothelial cells, and (*ii*) angiogenesis, which begins at the end of the fourth week of gestation and where the first vascular plexus are expanded and remodeled (Charnock-Jones et al., 2004; Gutiérrez et al., 2016). This process is finely tuned and regulated by different angiogenic factors including the vascular endothelial growth factor (VEGF), placental growth factor (PlGF), angiopoietins (ANG), fibroblast growth factor 2 (FGF2), and the insulin/insulin-like growth factors (INS/IGF) system (Burton et al., 2010; Burton and Jauniaux, 2015). Expression of these factors is highly regulated throughout gestation and is mainly attributed to trophoblast cells, Hofbauer cells, and smooth muscle cells, (Chen and Zheng, 2014). Since expression of angiogenic factors as well as the angiogenic process itself are under regulation by glycaemia, insulin, and hypoxia (Hadjipanayi and Schilling, 2013; Brocato et al., 2014; Chen and Zheng, 2014; Cvitic et al., 2014), vasculogenesis and angiogenesis processes at the fetoplacental vasculature are susceptible to alterations by a diabetic environment, such as in GDM or pregestational diabetes mellitus. Besides activation of the angiogenic factors, activation of ERS and UPR pathways (Paridaens et al., 2014) and dyslipidaemia (Oh et al., 2016), are also involved in physiological and pathological angiogenesis involving these molecules in the human placenta.

### Angiogenesis in GDM

**Figure 2:**
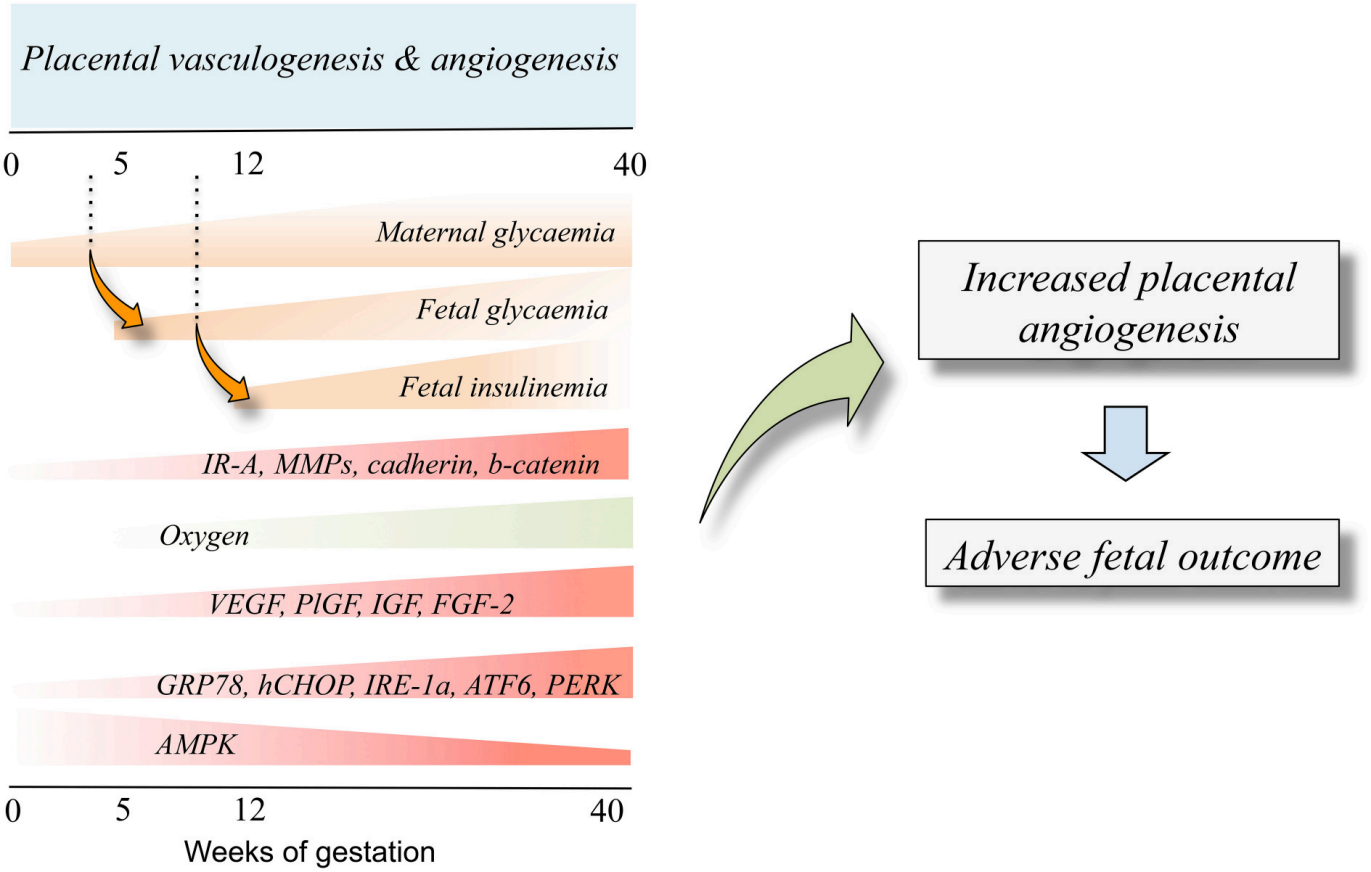
**Fetal insulinemia and altered angiogenesis in fetoplacental endothelium from gestational diabetes mellitus**. With the progression of pregnancy up to the 40th weeks of gestation, the maternal glycaemia increases, and could reach supraphysiological levels in pregnancies where the mother is diagnosed with gestational diabetes mellitus. The maternal hyperglycaemia results in increased fetal glycaemia from about the 5th week of gestation (dotted line), a condition resulting in a supraphysiological increase of fetal insulinemia from the 12th week of gestation. Increased fetal insulinemia results in altered placental vascular development and growth leading to angiogenesis alterations (*Placental vasculogenesis* and *angiogenesis*). Thus, an adverse fetal outcome is seen as a result of abnormal angiogenesis. Cell signaling mechanisms involved in this phenomenon include altered expression and/or activity of several molecules that are responsive to insulin (IR-A, MMPs, cadherin, b-catenin). Equally, a low oxygen level at the beginning of pregnancy increases the expression of proangiogenic growth factors (VEGF, PlGF, IGF, FGF-2) and increased (GRP78, CHOP, IRE-1α, ATF6, PERK) or reduced (AMPK) expression and/or activity. Composed from information reported by Babawale et al. (2000), Jirkovská et al. (2002), Easwaran et al. (2003), Baumüller et al. (2015), Westermeier et al. (2015b).

Placenta hypervascularization in women with DMT1, DMT2, or GDM, is reported (Cvitic et al., 2014; Huynh et al., 2015; Jarmuzek et al., 2015). DMT1 and DMT2 affect the entire process (vasculogenesis and angiogenesis; Jirkovská et al., 2002; Nelson et al., 2009; Jarmuzek et al., 2015) while GDM seems to impact the microvascular remodeling at angiogenesis (Jarmuzek et al., 2015). At 3rd trimester of pregnancy, the effect of DMT1, DMT2, and GDM on these phenomena is similar resulting in increased branching and surface area of villous capillaries (Teasdale, 1981; Jirkovská et al., 2002). Since GDM associates with developing longer umbilical cords compared with normal pregnancies (Georgiadis et al., 2014), it is suggested that placental hypervascularization in diabetes mellitus is mainly attributed to increased angiogenesis (Jirkovská et al., 2002; Leach, 2011; Figure 2). The later is partially explained by a placental hypoxia condition resulting from the fetal hyperglycaemia in diabetes mellitus. Fetal hyperglycaemia triggers fetal hyperinsulinemia, over-activating fetal metabolism leading to increased oxygen demand (Hytinantti et al., 2000; Taricco et al., 2009; Jarmuzek et al., 2015). Thus, it is likely that fetal hypoxia promotes the expression of angiogenic factors during the physiological placental angiogenesis at the 1st trimester of pregnancy when the oxygen level is reduced (Jauniaux et al., 2000). Since the level of FGF-2 is also regulated by hypoxia (Wang et al., 2009; Seo et al., 2013) and is increased in both the placenta and umbilical cord blood in diabetic pregnancies (Arany and Hill, 1998; Grissa et al., 2010), this growth factor emerges as a candidate to explain the hypervascularization in placentas from diabetes mellitus. Knowing that insulin is an angiogenic factor in endothelial cells (Liu et al., 2009), fetal hyperinsulinemia would have profound effects on placental and fetal vascular changes associated with maternal diabetes mellitus in pregnancy (Lassance et al., 2013). Interestingly, in another set of studies VEGF expression was shown to be lower in human placentas likely due to increased expression of the ERS-maker GRP78 (Aditiawarman, 2014). Thus, responses of ERS to a stressor will also result in an altered synthesis and/or release of proangiogenic factors in the human placenta vascular bed. This information is complemented by the proposed role of IRE1α, ATF6, and PERK as sensors of ERS (Marciniak and Ron, 2006; Zhang and Kaufman, 2006; Ron and Walter, 2007) or UPR (Ghosh et al., 2010; Hetz et al., 2015) constituting potential novel upstream regulatory pathways of angiogenesis via modulation of VEGF transcription in the human placenta (Iwawaki et al., 2009). Whether insulin modulates angiogenesis in the human placenta via changes in expression and/or function of ERS markers is unknown.

### Insulin, angiogenesis, and ERS in GDM

Insulin stimulates the formation of new blood vessels *in vivo* (Martínez-Jiménez et al., 2013), stimulates the formation of longer and branched blood vessels (Liu et al., 2009), and promotes microvascular endothelial cells migration (Liu et al., 2009). Thus, fetal hyperinsulinemia in GDM pregnancies could result in enhanced branching angiogenesis (Jirkovská et al., 2002) stimulating endothelial cells proliferation. This phenomenon is likely mediated by activation of IRs isoforms, which are expressed at villus branching spots in this cell type in the process of angiogenesis. At 1st trimester of pregnancy the IRs are mainly expressed by the syncytiotrophoblast, and in a lesser extent by cytotrophoblasts; however at term, IRs are mainly expressed in the fetoplacental vasculature (Desoye et al., 1994, 1997; Hiden et al., 2006). Thus, it is feasible that insulin regulates fetoplacental angiogenesis, but not, or in a minor degree, placental vasculogenesis at late pregnancy (Figure 2). Interestingly, mothers with GDM that were under insulin therapy show increased metalloproteases activity, probably mediated by activation of IR-A isoform. This is potentially due to higher levels of insulin-like growth factor 2 (IGF-2) as reported in placental endothelial cells from normal pregnancies (Hiden et al., 2012). Thus, IR-A is likely involved in proangiogenic pathways in GDM. On the other hand, placental histology in women with GDM that were under insulin therapy and show normal glycaemia at 3rd trimester of pregnancy, a decrease in the proangiogenic factors cadherin and b-catenin was reported (Babawale et al., 2000; Easwaran et al., 2003). Thus, GDM associates could course with impaired placental barrier leading to angiogenesis. However, the latter is contrary to the reported increase in expression of these molecules in these patients (Baumüller et al., 2015), highlighting a potential stimulatory effect of insulin involving these adhesion molecules to lead angiogenesis in the placenta (Table 2).

**Table 2:** **Effect of insulin on angiogenesis in the human fetoplacental vasculature in GDM**.

**Tissue/cells**	**Effect of GDM on angiogenesis**	**Insulin effect**	**Receptor/molecule involved**	**References**
Placental tissue^*^	*n.r.*	Increase	TK, cadherin–catenin	Babawale et al., 2000
Placental tissue	Increase	Increase	–	Jirkovská et al., 2002
Placental tissue	Increase	Increase	–	Westgate et al., 2006
Placental tissue	Increase	Increase	–	Hiden et al., 2009
Placental villi	Increase	Increase	–	Calderon et al., 2007
Placental villi	Increase	Decrease	VEGFR2, VEGF	Pietro et al., 2010
HUVECs^*^	*n.r.*	Increase	HIF1α, VEGF-A	Treins et al., 2002
fpECs	Increase	Increase	PI3-K	Hiden et al., 2012

TK, tyrosine kinase signaling pathway; VEGF, vascular endothelial growth factor; VEGFR2, VEGF receptor 2; HIF1α, hypoxia inducible factor 1; VEGF-A, VEGF A, HUVECs, human umbilical vein endothelial cells; fpEC, feto-placental endothelial cells; PI3-K, phosphatidylinositol 3-kinase.

* Samples taken from women with GDM under treatment with insulin; n.r., not reported.

It is interesting to notice that the metformin, an insulin sensitizer currently used to treat DMT2, is also an activator of the AMP-activated protein kinase (AMPK), which was shown to inhibit ERS restoring endothelial cell dysfunction in high fat diet-induced obese mice (Cheang et al., 2014). Since AMPK is a molecule whose activation may result in reducing ERS-markers activation (particularly PERK), and its expression is low in a mice model of GDM (Yao et al., 2015), it is feasible that it is involved in the GDM-associated increase in placental angiogenesis. Interestingly, AMPK activity was also reduced in skeletal muscle in obese pregnant women with GDM (Boyle et al., 2014), complementing the observations regarding AMPK as a potential ERS-marker in mice GDM. Other studies have recently shown that atherogenesis is also a process that involves ERS-factors activation (GRP78 and CHOP) and endothelial dysfunction in rabbits (Kruzliak et al., 2015). In addition, treatment of HUVECs with low-density lipoprotein (LDL) activated UPR and interleukins expression (Gora et al., 2010). Certainly, further research is necessary in order to understand whether insulin treatment during pregnancy in women with GDM or coursing without or with supraphysiological hypercholesterolaemia (Leiva et al., 2015) leads to a beneficial or detrimental result on overall angiogenic mechanisms involving or not ERS and/or UPS pathways in the human fetoplacental vasculature.

## Maternal dyslipidaemia

Dyslipidaemia is defined as the elevated blood level of triglycerides (hypertriglyceridemia) and TCh (hypercholesterolaemia) including increased LDL and reduced high-density lipoprotein (HDL) levels [National Cholesterol Education Program (NCEP), 2002]. This pathological condition is recognized as the main risk factor for the development of cardiovascular disease [National Cholesterol Education Program (NCEP), 2002; Arsenault et al., 2011]. GDM also courses with maternal dyslipidaemia affecting fetal development and growth (Desoye and Hauguel-de Mouzon, 2007; Sanchez-Vera et al., 2007; Marseille-Tremblay et al., 2008; Schaefer-Graf et al., 2008). Indeed, hypercholesterolaemia was shown to contribute to endothelial dysfunction in the fetal vasculature in this disease (Reece, 2010; Sreckovic et al., 2014). Interestingly, GDM-associated increase in the maternal plasma lipids may result in abnormal transport of these molecules across the placenta into the developing fetus, a phenomenon likely regulated by insulin (Herrera and Desoye, 2015). Thus, alterations of a maternal lipids profile in GDM could lead to alterations in the fetal circulating level of lipids or to a defective composition of lipid macromolecules making HDL or other lipids less functional in the fetus (Leiva et al., 2015).

### Dyslipidaemia in GDM

**Figure 3:**
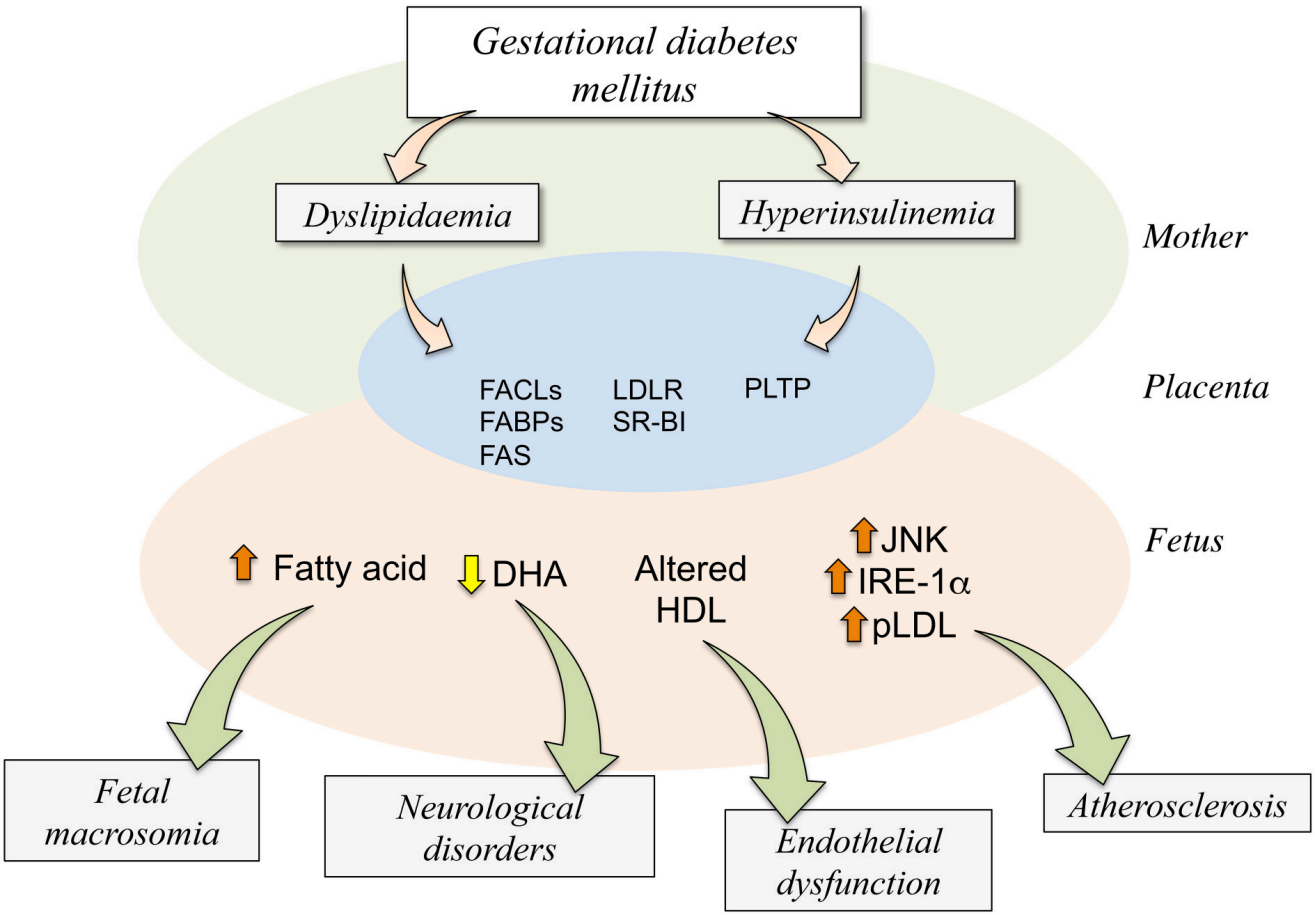
**Potential consequences of dyslipidaemia and hyperinsulinemia on the human fetoplacental unit from gestational diabetes mellitus**. Gestational diabetes mellitus (GDM) results in maternal (*Mother*) metabolic alterations leading to dyslipidaemia and hyperinsulinemia. These two abnormal metabolic conditions are associated with higher expression and activity of several molecules involved in the placental transport (FABPs,) and metabolism (FACLs, FAS, PLTP) of lipids or its receptors (LDLR, SR-BI) in the human trophoblast (*Placenta*). These changes result in altered transplacental transport of several signaling molecules via the trophoblast barrier, ending in increased (⇧) fatty acid, reduced (⇩) docosahexaenoic acid (DHA), or altered composition or function of high-density lipoprotein (HDL) in the fetoplacental circulation (*Fetus*). Since these changes in the capacity of transport by the placenta increased levels of endoplasmic reticulum stress (JNK, IRE-1α) and atherosclerotic [phospholipolyzed LDL (pLDL)] markers are detected in the fetal endothelium. Adverse fetal outcome results from alterations in the fetal circulating or tissue levels of these molecules in GDM compared with normal pregnancies. Increased level of fatty acids regards with a higher incidence of fetal macrosomia while a decrease in the fetal plasma level of DHA associates with increased number of neurological disorders. Additionally, less functional HDL could potentially result in endothelial dysfunction in the newborn, and atherosclerosis could result from increased ERS markers. Composed of information reported by Ethier-Chiasson et al. (2007), Marseille-Tremblay et al. (2008), Herrera and Ortega-Senovilla (2010), Scifres et al. (2011), Olmos et al. (2012), Pagán et al. (2013), Araújo et al. (2013), Sreckovic et al. (2013), Herrera and Desoye (2015).

GDM courses with increased maternal TCh and triglycerides altering the expression and function of proteins involved in triglycerides and cholesterol homeostasis (Marseille-Tremblay et al., 2008; Radaelli et al., 2009; Herrera and Ortega-Senovilla, 2010; Herrera and Desoye, 2015). These changes include increased expression of genes related to lipid transport and metabolism such as the fatty acyl-CoA ligases (FACLs), which catalyse conversion of fatty acids into fatty acyl-CoA esters required for the synthesis of triglycerides, increased cholesterol and membrane phospholipids, higher expression and activity of placental fatty acid binding proteins (FABPs), endothelial and lipoprotein lipases that favor the breakdown of maternal triglycerides into fatty acids (Radaelli et al., 2009; Figure 3). Increased FABPs found in GDM pregnancies leads to binding fatty acids from the maternal circulation to export these to the fetal circulation. FACLs could favor the synthesis of triglycerides in the fetal circulation, and endothelial lipases and lipoprotein lipases could increase the breakdown of maternal triglycerides favoring the uptake of the fatty acids by the trophoblast. Additionally, placental expression of fatty acid synthase (FAS) is increased in placentas from GDM (Marseille-Tremblay et al., 2008), suggesting that lipid metabolism in the placenta is altered by this pathological condition. Interestingly, in placental tissue and trophoblast from GDM a higher level of lipid droplets has been reported, suggesting that lipid content is higher in this pathological condition compared with normal pregnancies (Elchalal et al., 2005; Scifres et al., 2011).

GDM effect on lipoprotein receptors expression has also been reported. Whereas, in normal pregnancies maternal hypercholesterolaemia associates with lower expression of LDL receptor (LDLR) in homogenized placenta (Ethier-Chiasson et al., 2007; Desoye et al., 2011), expression of the HDL scavenger receptor class B type I (SR-BI) and LDLR is increased in GDM compared with normal pregnancies (Dubé et al., 2013). Thus, GDM affects maternal and neonatal lipid profiles perhaps predisposing the fetus to future metabolic diseases (Dubé et al., 2013). Another protein involved in the lipoprotein metabolism that is also modified by GDM in placental cells is the phospholipid transfer protein (PLTP), which is involved in the metabolism of fetal HDL and directly related with the HDL remodeling leading to a larger HDL molecule (Tzotzas et al., 2009). PLTP is expressed in endothelial cells of the placental vasculature (Marceau et al., 2005; Scholler et al., 2012a). GDM associates with upregulation of PLTP in the placental endothelium (Scholler et al., 2012b), a phenomenon due to the hyperinsulinemia and hyperglycaemia. PLTP increased expression could also be a phenomenon associated with the increased concentration of HDL described in newborns from GDM pregnancies (Merzouk et al., 2000; Scholler et al., 2012a,b; Sreckovic et al., 2014).

### Insulin, dyslipidaemia, and ERS in GDM

In normal and GDM pregnancies transfer of lipids from the maternal to the fetal blood across the placenta is a process highly regulated by the level of insulin (Table 3). Interestingly, incubation of trophoblast cells from normal pregnancies with insulin and fatty acids (i.e., concomitant conditions in GDM) increase lipid droplets formation, suggesting that trophoblast is involved in packaging lipids. The mechanisms involved in this phenomenon include insulin-stimulated overexpression of adipophilin (a protein involved in fatty acid uptake and storage in adipocytes; Elchalal et al., 2005). However, human trophoblast from GDM pregnancies shows higher expression of FABPs isoform 4 (FABP4), which was suggested as likely responsible for the associated increase in placenta lipid droplets (Scifres et al., 2011). However, increased FABP4 expression and lipid droplets formation are not regulated by insulin in these cells. Thus, insulin-modulated mechanisms involved in lipid droplets formation are likely to be different in normal compared with GDM pregnancies. Other findings show that GDM-associated reduction in the mother-*to*-placenta transfer of the fatty acid docosahexaenoic acid (DHA) is worsened in mothers with GDM under insulin therapy (Pagán et al., 2013; Larqué et al., 2014; Sobrevia et al., 2015). Interestingly, DHA uptake is increased by insulin in human trophoblast from normal pregnancies (Araújo et al., 2013). Thus, a potential increase in DHA uptake is unlikely in trophoblast from GDM pregnancies since plasma insulin in the fetoplacental circulation is higher in GDM compared with normal pregnancies (~75 vs. ~40 pmol/L; Westermeier et al., 2011, 2015a; Salomón et al., 2012; Guzmán-Gutiérrez et al., 2016). HDL metabolism at the fetal circulation is also altered in GDM pregnancies mainly due to upregulation of PLTP expression and activity (Scholler et al., 2012a; Sreckovic et al., 2014). Since insulin increases this protein expression in human placental endothelium, it is feasible that this hormone contributes to the synthesis of a larger HDL molecule as seen in GDM as well as with the increase in maternal-*to*-fetal cholesterol transfer (Scholler et al., 2012a,b). Worryingly, increased fatty acids or decreased DHA in the fetal circulation under maternal dyslipidaemia and hyperinsulinemia associates with fetal macrosomia (Herrera and Desoye, 2015) and neurological disorders (Araújo et al., 2013; Larqué et al., 2014; Figure 3).

**Table 3:** **Effect of insulin on the expression of molecules involved in lipids metabolism in GDM**.

**Cells/tissue**	**Molecule**	**GDM effect on expression**	**Physiological consequences in GDM**	**Insulin effect**	**References**
HPECs	PLTP	Increased	Increase in fetal HDL level	Decrease	Scholler et al., 2012b
PHT	FABP4	Increased	Increase in placental lipid droplets formation	Unaltered	Scifres et al., 2011
PHT	ACSL	Reduced	Reduced DHA uptake	Increase	Araújo et al., 2013
PHT	ACSL	Reduced	Reduced AA uptake	Unaltered	Araújo et al., 2013
Placental blood	*n.r.*	*n.r.*	Reduced DHA in the placenta blood	Decrease	Larqué et al., 2014
Placental blood	*n.r.*	*n.r.*	Reduced DHA in the placenta blood	Decrease	Pagán et al., 2013
Fetal blood	*n.r.*	*n.r.*	Reduced DHA in the fetal blood	Decrease	Larqué et al., 2014
Fetal blood	*n.r.*	*n.r.*	Reduced DHA in the fetal blood	Decrease	Pagán et al., 2013
Placenta	HADHA	Reduced	*n.r.*	*n.r.*	Austin et al., 2004
Placenta	AGPAT2	Reduced	*n.r.*	*n.r.*	Austin et al., 2004

HPECs, human placental endothelial cells; PHT, primary human trophoblasts; PLTP, phospholipid transfer protein; FABP4, fatty acid binding protein 4; ACSL, long-chain acyl-CoA synthetase; DHA, docosahexaenoic acid; AA, araquidonic acid; HDL, high-density lipoprotein; n.r., not reported.

Modified lipoproteins profile has also been associated with ERS pathway as previously reviewed (Lenna et al., 2014). In brief, it has been suggested that oxidized LDL (oxLDL) may cause atherosclerosis requiring JNK and IRE-1α activation, thus involving ERS and UPR pathways in this phenomenon (Sanson et al., 2009). In addition, the pro-inflammatory phospholipolyzed low-density lipoprotein, which is increased in atherosclerotic lesions, activated UPR and interleukins expression in the treatment of HUVECs (Gora et al., 2010). Thus, fetoplacental endothelial cells are prone to activate ERS, and perhaps URP pathways in maternal dyslipidaemia. However, nothing is reported on the potential effects of insulin and the involvement of IRs isoforms in this phenomenon.

## Concluding comment

**Figure 4:**
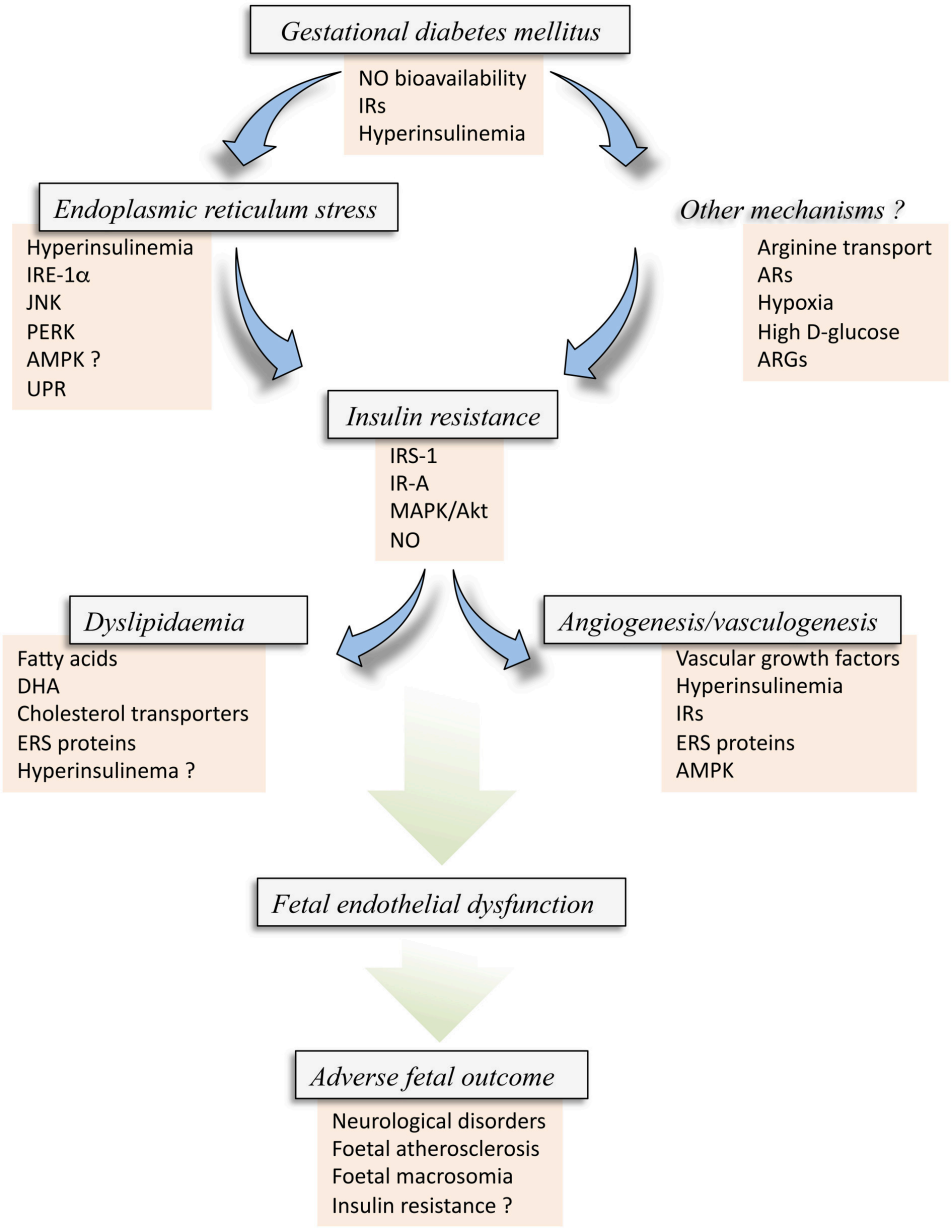
**Insulin resistance in the fetus from gestational diabetes mellitus**. Gestational diabetes mellitus (GDM) is a disease coursing with fetal hyperinsulinemia and associated with metabolic alterations that result in reduced bioavailability of nitric oxide (NO) and altered expression of insulin receptors (IRs). These alterations, and perhaps hyperinsulinemia itself, may result in abnormal expression and activity of several molecules associated with endoplasmic reticulum stress (ERS) [IRE-1α, JNK, PERK, and likely AMPK (AMPK?)], which could also lead to the activation of the unfolded protein response (UPR) pathway. ERS via this set of alterations could result in *Insulin resistance* in the fetus/newborn. Insulin resistance could alternatively be caused by *Other mechanisms*, including membrane transport of L-arginine, the substrate for NO synthesis, adenosine receptors (ARs) expression and/or activation, hypoxia or high extracellular concentration of D-glucose, and arginases (ARGs) activity, a metabolic pathway that consume L-arginine in endothelial cells. All these factors could result in increased inhibitor phosphorylation of IRS-1, perhaps involving the subtype A of IRs (IR-A) with a deficient signaling pathway mediated by p44/42^mapk^ (MAPK) and protein kinase B/Akt. These phenomena lead to reduced synthesis and/or bioavailability of NO in the endothelial cells from the human placenta. Since insulin signaling is crucial maintaining a normal metabolism of lipids and angiogenesis and vasculogenesis in the human placenta from normal pregnancies, GDM-associated fetal insulin resistance could result in altered mother-*to*-fetus transplacental transfer due to altered expression and activity of cholesterol transporters, and metabolism of lipids leading to accumulation of fatty acids, docosahexaenoic acid (DHA), or cholesterol. A generalized metabolic disturbance referred as *Dyslipidaemia*. The latter is also associated with increased expression of ERS markers, and could also be due to hyperinsulinemia (Hyperinsulinemia ?). Additionally, insulin resistance results in a lack of modulation of physiological *Angiogenesis* and *vasculogenesis* in pregnancy where and increase in vascular growth factors, hyperinsulinemia, IRs and ERS molecules, including AMPK activity, are potentially involved. All these phenomena, i.e., GDM, ERS, angiogenesis and dyslipidaemia, develop with altered expression and activity of common molecules due to the state of insulin resistance in the fetoplacental vasculature. The final result is an abnormal function of the fetal endothelium (*Fetal endothelial dysfunction*) that ends with *Adverse fetal outcome* characterized by increased risk of fetal/newborn atherosclerosis, macrosomia, neurological disorders, and insulin resistance.

Altered vascular function in GDM pregnancies is a critical condition leading to severe dysfunction of the human placenta and altered delivery of nutrients and signaling molecules from mother-*to*-fetus and vice-versa (Desoye et al., 2011; Leach, 2011; Herrera and Desoye, 2015; Sobrevia et al., 2015). Several mechanisms leading to abnormal function of the human placenta regards with metabolic alterations of this organ, including ERS (Lenna et al., 2014) and metabolism of lipids (Leiva et al., 2015), as well as metabolic-derived structural modulation of the placental vascular bed, such as angiogenesis and vasculogenesis (Gutiérrez et al., 2016; Figure 4). Interestingly, GDM leads to a state where cell signaling mechanisms associated with insulin biological effects in cells from the fetoplacental vasculature, and perhaps in other vascular beds, is altered leading to a potential state of insulin resistance (Colomiere et al., 2009; Westermeier et al., 2015b). One of these metabolic conditions is ERS where key molecules (e.g., ISR-1, JNK, IRE-1α, and potentially AMPK) are apparently involved. Abnormal insulin signaling in the human fetoplacental endothelium results in a lower NO bioavailability and Akt activation, thus reducing fetoplacental vascular reactivity *in vitro*. These phenomena could explain the abnormal or even lack of regulation of a vectorial mother-*to*-fetus transplacental transport of nutrients, which could result in altered fetal growth and development, with subsequent consequences at birth and/or adulthood.

Reduced NO synthesis could also be a condition leading to abnormal angiogenesis (Gutiérrez et al., 2016). Interestingly, fetal hyperinsulinemia in GDM pregnancies results to be a key factor leading to an abnormal fetal outcome, including macrosomia (Olmos et al., 2012; Leiva et al., 2015) and endothelial dysfunction (Sobrevia et al., 2015). The possibility of a reduced vascular reactivity to insulin in GDM pregnancies is likely and it is a phenomenon that could explain the diminished response of the fetoplacental vascular endothelium to this hormone. Even when women coursing with GDM pregnancies that do not reach normal glycaemia by diet/exercise are passed into insulin therapy [Verier-Mine, 2010; American Diabetes Association (ADA), 2015; Sobrevia et al., 2015], it is unknown whether treatment with insulin in this group of women, which in fact normalizes their glycaemia, will result in normalization of the microvascular and macrovascular fetoplacental endothelial function.

In summary, we propose insulin as a key factor playing a modulatory role in GDM-associated altered angiogenesis, ERS, and metabolism of lipids in the human fetoplacental vascular bed. Since GDM courses with fetoplacental insulin resistance state at birth, the potential beneficial effect of this hormone on these phenomena is restricted. The possibility that clinical management of insulin sensitivity is considered a therapeutic target in the treatment of mothers with this disease could result in reversing along with insulin resistance, the GDM-associated alterations in ERS, angiogenesis and lipids metabolism. However, we emphasize to take with caution the broad spectrum of results reported in primary cell cultures, cell lines, or experimental models, as summarized in this review, so not to extrapolate the findings to what is happening in the mother and their newborn in GDM. Certainly, a better understanding of the mechanisms behind these alterations caused by GDM, including pregnant women with this disease but treated with diet/exercise or under insulin therapy, and the potential effect of insulin in this phenomenon, is crucial in the aim of preventing adverse fetal outcome from this disease of pregnancy.

## Author contributions

Conception and designed of the manuscript: LS, AL, MF, JG, IC, FP. Acquisition of data/information: RS, BF, EB, CP, LT, FT, MG, RV, MS, JA, LSi, IC. Analysis of data/information: LS, AL, MF, JG, FP, FT, RS, EB, BF. Interpretation of data/information: LS, AL, MF, JG, FP, LT, RS, MG, BF, EB. Compilation of tables: RS, BF, LT, CP, LS, AL, FP. The design of figures: LS, AL, JG, FP.

## Funding

This work was supported by Fondo Nacional de Desarrollo Científico y Tecnológico (FONDECYT 1150377, 1150344, 1121145, 11150083), VRID-Asociativo project (213.A84.014-1.0), Universidad de Concepción, and Dirección de Investigación, Universidad San Sebastián, Chile. RS, LS, RV, and MS hold a Comisión Nacional de Investigación en Ciencia y Tecnología (CONICYT) Chile–Ph.D. fellowship. RS, BF, LS, and EB hold Faculty of Medicine, PUC–Ph.D. fellowships. RV and MS hold Vicerectorate of Research, PUC-Ph.D. fellowships.

### Conflict of interest statement

The authors declare that the research was conducted in the absence of any commercial or financial relationships that could be construed as a potential conflict of interest.
